# Tribotronic bipolar junction transistor for mechanical frequency monitoring and use as touch switch

**DOI:** 10.1038/s41378-018-0026-1

**Published:** 2018-11-05

**Authors:** Fengben Xi, Yaokun Pang, Wenjian Li, Tianzhao Bu, Junqing Zhao, Guoxu Liu, Tong Guo, Wenbo Liu, Chi Zhang

**Affiliations:** 1 CAS Center for Excellence in Nanoscience, Beijing Key Laboratory of Micro-nano Energy and Sensor, Beijing Institute of Nanoenergy and Nanosystems, Chinese Academy of Sciences, 100083 Beijing, China; 2 School of Nanoscience and Technology, University of Chinese Academy of Sciences, 100049 Beijing, China

## Abstract

Tribotronics, a new field that involves the coupling of triboelectricity and semiconductors, has attracted great interest in the nanoenergy and nanoelectronics domains. This paper proposes a tribotronic bipolar junction transistor (TBJT) that incorporates a bipolar junction transistor and a triboelectric nanogenerator (TENG) in the single-electrode mode. When the mobile triboelectric layer slides on the device surface for electrification, a bias voltage is created and applied to the emitter junction, and then the base current from the TENG is amplified. Based on the fabricated TBJT, a mechanical frequency monitoring sensor with high sensitivity and excellent stability and a finger-triggered touch switch were developed. This work demonstrated for the first time a tribotronic device with simultaneously controlled voltage and current voltage/current simultaneously controlled tribotronic device, which has promising potential applications in micro/nano-sensors, human-machine interactions, intelligent instrumentation, wearable electronics, and other applications.

## Transistors: Triggered by touch

A new type of transistor that is switched by movement could greatly enhance interactions between machines, humans and the environment. The transistor, developed by Chi Zhang and co-workers at the Chinese Academy of Sciences in Beijing, takes advantage of triboelectricity – the electrification of objects by friction, such as when a balloon is rubbed on clothes. Triboelectricity has recently enabled researchers to convert mechanical energy into electricity in nanoscale devices, but not in bipolar junction transistors until now. Zhang and co-workers connected a copper pad to the base input of a silicon transistor structure, and showed that they could switch the transistor on or off by either lifting or sliding a polymer film across the copper pad. The team have already used their ‘tribotronic’ transistor design to develop highly sensitive mechanical frequency sensors and a finger-triggered touch switch.

## Introduction

In the last few decades, transistor scaling has followed the well-known Moore’s law, resulting in an increase of two times in the transistor density every 2 years^1,2^, which is approaching the limitations of physical size and power^3,4^. Therefore, information technology (IT) in the post-Moore’s law period will develop toward new directions including diversification, improved sensors, multifunctionality, and self-powering capacities^5–10^. As one of the basic transistors, the bipolar junction transistor (BJT) still plays an important role in the design of discrete and very-high-frequency circuits and is used as an amplifier or switch because of its high transconductance and output resistance compared to MOSFETs^11,12^. However, the traditional BJT is modulated by internal electrical signals and lacks the direct interaction mechanism between the external environment and electronics.

Recently, the invention of a triboelectric nanogenerator (TENG) has successfully provided an effective approach to convert ambient mechanical energy into electricity^13^. The working principle of the TENG is based on contact electrification and electrostatic induction, which have been widely used in micro/nano-energy^14–17^, self-powered systems^18–26^, and blue energy^27–29^. Furthermore, by coupling the triboelectricity with semiconductors, a new field of tribotronics has been proposed^30–32^, which concerns research regarding the interaction between triboelectricity and semiconductors^33^ using triboelectric potential controlling electrical transport and transformation in semiconductors for information sensing and active control^34–36^. To date, various tribotronic devices have been developed including contact-gated LEDs^37^, touch memory^38^, adjustable phototransistors^39,40^, sensing arrays^41^, tribotronic tuning diodes^42^, and flexible organic tribotronic transistor^43^. All these applications demonstrate the potential of tribotronics in active interaction electronics, offering a prospective strategy to design smart sensors with the advantages of low cost, simple mechanism, and excellent sensitivity.

In this study, we developed a tribotronic BJT (TBJT) by combining a triode with a TENG in the single-electrode mode. The collector current of the TBJT can be amplified and tuned by the base current from the TENG with the sliding motion of the mobile triboelectric layer. Based on the fabricated TBJT, a mechanical frequency monitoring sensor and a finger-triggered active smart touch switch have been developed. In contrast, from previous tribotronic transistors, the TBJT is controlled by the voltage and current created by the TENG simultaneously, which has potential applications in micro/nano-sensors, human-machine interaction (HMI), intelligent instrumentation, and remote controls.

## Results

### Structure of the TBJT

**Fig. 1:**
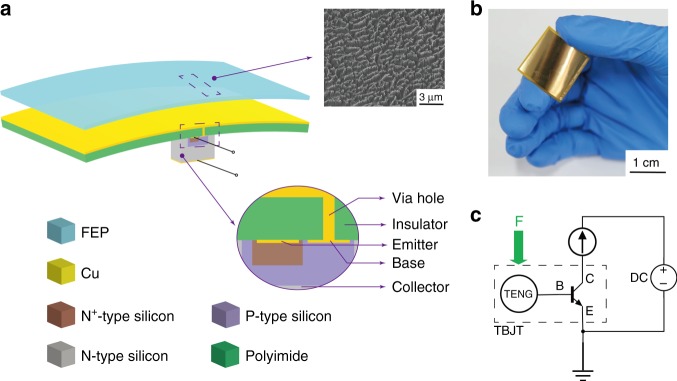
Basic structure of the tribotronic bipolar junction transistor (TBJT). **a** Schematic illustration of the TBJT. Inset: cross-section configuration of the TBJT, showing an electrical connection between the TENG in the single-electrode mode and an n-p-n bipolar junction by via hole; SEM image of nanostructures on the surface of the mobile triboelectric layer made of FEP film. **b** Optical photograph of the TBJT. **c** Equivalent circuit of the TBJT

The basic structure of the TBJT comprises a flexible polyimide substrate, a Cu pad (25 mm × 25 mm), a free-standing fluorinated ethylene propylene (FEP) film, and an NPN type triode, as schematically illustrated in Fig. 1a. The Cu pad is deposited on the top surface of the flexible polyimide substrate, and the triode made of a silicon-based n-p-n junction is constructed on the bottom layer. The emitter, base and collector are electrically connected with the n + -type, the p-type, and the n-type region of the triode, respectively. Through the designed via hole, the Cu pad is also electrically connected to the base electrode, as shown in the inset of the cross-section. The mobile layer is assembled next to the Cu pad, which experiences vertical contact and separation from the Cu pad by the external force. The other inset shows the SEM image of the nanostructures on the surface of the mobile triboelectric layer made of FEP film, which is modified via inductive coupling plasma (ICP) to enhance the surface triboelectric charge density. A well-designed TBJT is presented in Fig. 1b (the detailed fabrication process is introduced in the Methods section). Figure 1c presents the equivalent circuit of the TBJT, which intuitively shows the interaction between the external force and the electronics.

### Working mechanism of the TBJT

**Fig. 2:**
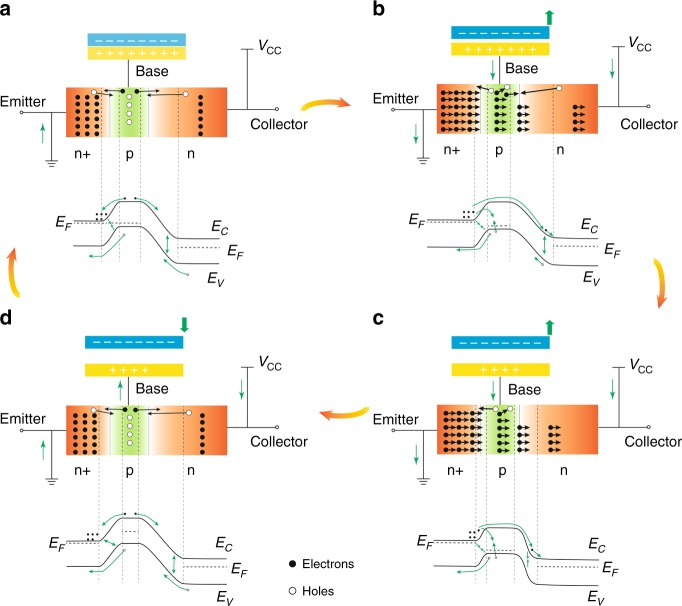
Proposed working mechanism of the TBJT and band structures of the TBJT at four positions. **a** When the FEP film comes in contact with the Cu pad, the TBJT and band diagram are in the cut-off mode. **b** The TBJT and band diagram are in the active mode when the FEP film moves the surface slightly away from the Cu pad. **c** When the FEP film is moved sequentially, the TBJT and band diagram are in the saturation mode. **d** The TBJT and band diagram return to the cut-off mode when the FEP electrification film slides backwards

The working mechanism of the TBJT is elaborated in Fig. 2, which demonstrates a working principle different from that of the conventional BJT configuration, based on the coupling of the NPN-BJT, triboelectrification and electrostatic induction. As demonstrated in Fig. 2a, the FEP film is purposely chosen as the mobile triboelectric layer owing to its high electronegativity according to the triboelectric series^44^. The collector of the TBJT is connected with a voltage source, whereas the emitter is grounded.

In the initial position, when the FEP film fully contacts the Cu pad, equal negative and positive charges are induced on the surfaces of the FEP film and the Cu pad, respectively, owing to their difference in triboelectric polarity. Because the positive triboelectric charges completely balance out the negative counterpart in this circumstance, there is no electrical potential difference applied to the base region and no charge transfer occurs from the TENG. The emitter–base junction is zero offset and the collector–base junction is reverse biased; therefore, the TBJT stays in the cut-off mode, which corresponds to a logical “off” state. Meanwhile, the depletion regions are generated in both the junctions where the electrons and holes recombine. Moreover, the Fermi levels of the base and emitter region in Fig. 2a are horizontally aligned, and are higher than those of the collector in its band diagram.

Once the FEP film is gradually separated from the Cu surface as the external force is vertically applied, the equilibrium potential is disturbed, resulting in a positive electrical potential being applied to the triode (Fig. 2b). Meanwhile, the emitter–base junction is forward biased (the depletion layer width decreases) and the collector–base junction is reverse biased (the depletion layer width increases); thus, the TBJT shifts into the active mode. In the active mode, the electrons are injected from the forward biased n + -type emitter region into the p-type base where they diffuse as minority carriers to the reverse-biased n-type collector and are swept away by the electric field in the reverse-biased collector–base junction. Then, based on the law of charge conservation, the current from the TENG is amplified dramatically in the collector by the triode. The Fermi level of the base region in Fig. 2b decreases owing to the positive potential in the base. Next, as shown in Fig. 2c, when the movement of the FEP film continues, the inner electric field gradually increases as well. Therefore, with both junctions being forward-biased (widths of both depletion layers decrease), the TBJT enters the saturation mode, and the Fermi level of the base region decreases between the emitter and collector. This mode corresponds to a logical “on,” or a closed switch, which means that the collector current has a high magnitude and is stable. Finally, the TBJT and its band diagram return to the cut-off mode when the FEP film slides backwards (Fig. 2d). However, in this process, the Fermi level of the base region increases because of the negative potential in the base.

Figure S1 shows the theoretically calculated results (performed by the software Comsol Multiphysics) of the potential difference generated from the contact-separating and sliding mode TENG with the FEP and copper films, indicating that this triboelectric potential varies with the separation distance of the mobile triboelectric layer.

### Electrical characterization of the TBJT

**Fig. 3:**
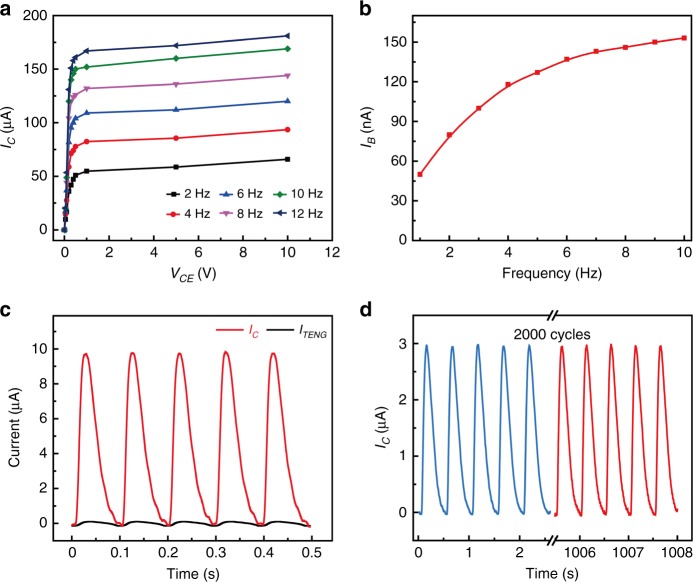
Electrical characterization of the TBJT. **a** Output characteristic curves of *V*_CE_–*I*_C_ with different frequencies. **b** Relationship between the *I*_B_ and the frequency. **c** Amplification curve of collector current and base current at the frequency of 4 Hz and *V*_CE_ = 0.5 V. **d** Stability test of the sensor. *V*_CE_ remains at 0.5 V during the entire experiment. A small hysteresis and good repeatability are attained even after tests of ~2000 cycles

To better evaluate the performance of the TBJT, its electrical characterizations were systematically studied, as depicted in Fig. 3. The measured results demonstrated satisfactory performance of the BJT. Figure 3a plots the output characteristic of the TBJT, and the separation frequency of the FEP film can be accurately controlled using a linear motor. As shown in the graph, the *I*_C_ rises as the separation frequency increases from 2 Hz to 12 Hz within a *V*_CE_ of 0–10 V. The curves of *I*_B_ and frequency were also studied, and are shown in Fig. 3b. When the separation frequency of the FEP film varies from 0 to 10 Hz, the *I*_B_ increases from 50 to 153 nA. In addition, the curve of the collector current and base current at the frequency of 4 Hz and *V*_CE_ = 0.5 V are shown in Fig. 3c, and the amplification factor of the peak current (*I*_C_ = 9.7469 μA and *I*_TENG_ = 0.0974 μA) can be reached at 100 × . Moreover, measurements for ~2000 cycles are carried out to validate the stability and repeatability of the device, as described in Fig. 3d. Even after 2000 test cycles, the changes in the peak collector current *I*_C_ are <5%, showing its small hysteresis and excellent reproducibility. The above experimental results indicate that the collector current increases with the increasing frequency, which corresponds to good electrical characterization. Owing to the direct interaction mechanism between the external environment and electric device, the TBJT is likely to have excellent applications in sensors and HMI.

## Discussion

### Application of the TBJT for mechanical frequency monitoring

**Fig. 4:**
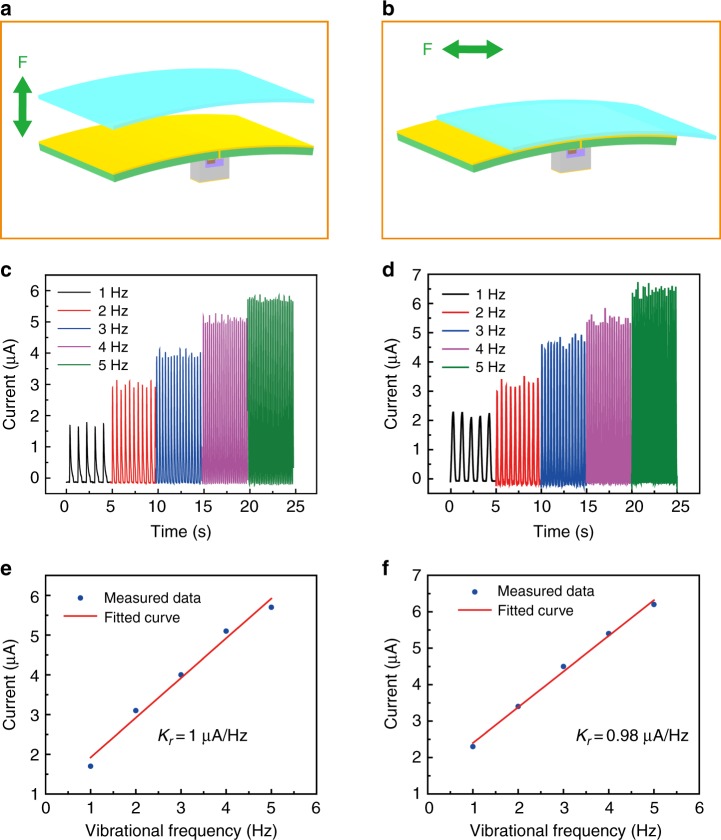
TBJT for signal frequency monitoring. **a**, **b** Schematic illustration of the device structure with contact-separating mode and sliding mode, respectively. **c** The collector current, when the FEP film vertically contacts and separates from the copper film with a frequency ranging between 1–5 Hz with collector voltage 0.5 V. **d** The collector current, when the FEP film and the copper film slide against each other with a frequency ranging between 1–5 Hz with collector voltage 0.5 V. **e** Relationship between the collector current and the vibration frequency; the sensitivity is 1.00 μA Hz^−1^. **f** Relationship between the collector current and sliding frequency; the sensitivity is 0.98 μA Hz^−1^

First, the TBJT was applied to mechanical frequency monitoring (Fig. 4). Based on the electrical characterization of the TBJT, the *I*_C_ can be tuned by the separation frequency of the FEP film. Based on this principle, we have designed a mechanical frequency monitoring sensor based on the TBJT. The structures of the TBJT with contact-separating and sliding single electrode modes, which are developed for vibrational and sliding frequency monitoring, are shown in Fig. 4a, b, respectively. Figure 4c–f show the characteristics of the mechanical frequency monitoring sensors. When the FEP film undergoes vertically reciprocating motion with the Cu film at a frequency from 1 to 5 Hz with a collector voltage of 0.5 V, the *I*_C_ increases with the increase in the vibrational frequency, as shown in Fig. 4c. The sensor exhibits good sensing performance with an exceptional sensitivity of 1.00 μA Hz^−1^. Similarly, as demonstrated in Fig. 4d, f, when the FEP film and the copper film slide against each other, there is a positive correlation between the *I*_C_ and the sliding frequency, and the sensitivity is 0.98 μA Hz^−1^. By exploiting the excellent sensitivity, the TBJT as a mechanical frequency monitoring sensor shows promising prospects in HMI and intelligent sensing.

### Application of the TBJT as a finger-triggered touch switch in smart home

**Fig. 5:**
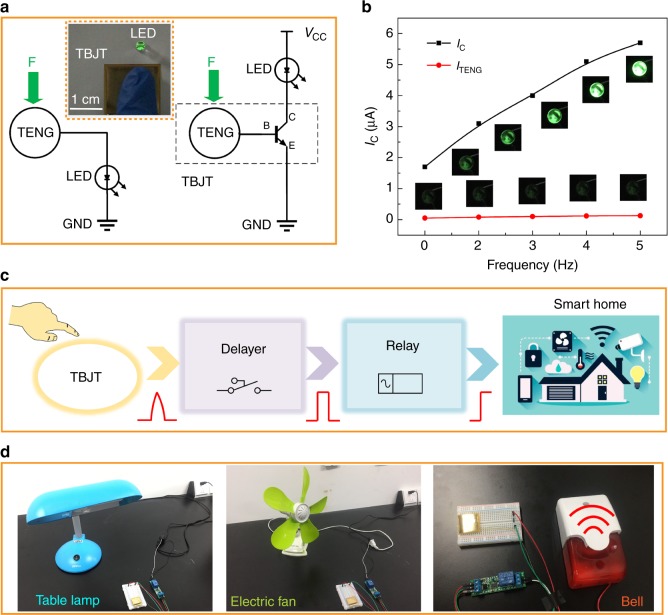
Application of the TBJT as a finger-triggered touch switch in smart home. **a** Equivalent electrical circuits of the TBJT and TENG for controlling the LED. Inset showing that an LED can be tuned directly by finger touch or release. **b** LED lights are tuned by the external mechanical force at frequencies of 0, 1, 2, 3, 4, and 5 Hz. **c** Schematic of a TBJT-involved home control system. After the finger touch and release, an impulse signal can be converted into a trigger signal to control the appliances. **d** Demonstration of the control of a table lamp, an electric fan, and a household security bell

In addition to being a mechanical signal sensor, another intriguing application of the TBJT is as a finger-triggered active tactile touch switch for smart home control systems. Traditional transistors cannot be touched by human fingers directly owing to the presence of electrostatic charges. However, on the basis of the strong advantages of the TBJT, we designed a finger-triggered active tactile switch with a high sensitivity that can be used in the fields of HMI and remote control. In this configuration, the human skin (finger) can also act as a mobile triboelectric layer, replacing the FEP film according to the triboelectric series. The equivalent electrical circuits of the TBJT and TENG used for controlling the light-emitting diode (LED) are demonstrated in Fig. 5a. The demo optical graph in the inset shows that an LED is controlled directly by the finger touching or releasing the mobile triboelectric layer (FEP film) of the TBJT. As depicted in Fig. 5b, the LED brightness can be tuned by the switch, and the driving energy of TENG is greatly improved. The LED is turned on/off when the finger presses or releases the switch, and the dynamic demonstrations are shown in Video S1.

Moreover, as shown in Fig. 5c, the smart home control system includes a person; a touch switch based on the TBJT; a simple signal processing circuit; and household appliances, such as a table lamp, an electric fan, and a household security bell. The signal processing module is made up of a delayer and a relay based on a single-chip microcomputer, and the detailed circuit board is demonstrated in Figure S2. When a user touches the Cu pad of the TBJT, the signal is detected and converted into a switching signal by the signal processing circuit for the household appliances (Fig. 3d and Video S2 to S4).

## Materials and methods

### Fabrication of the tribotronic bipolar junction transistor

First, a 100 μm polyimide (PI) substrate (28 mm × 28 mm) was prepared and cleaned in an ultrasonic cleaner with deionized water, ethanol, and acetone, sequentially, and then it was blow-dried in a drying oven at 100 °C for 1 h. Subsequently, three localized via holes were drilled on the substrate and 10 μm Cu used as electrical wires were deposited onto the selective area of back side of the substrate according to the designed circuit configuration. Next, layers of 3 μm Ni and 25 μm Cu were electroplated onto the substrate to form the pad (25 mm × 25 mm). Finally, a chip of the NPN-triode was adhered to the bottom of the PI substrate, in which the base was connected to the Cu pad through via the hole, and the collector and emitter could be electrically connected to the external circuitry by the electrical wires. The mobile layer vertically controlled by the external force was fabricated based on a piece of FEP film, which was also used as a mechanical frequency monitoring sensor.

### Surface modification of the FEP film

First, a 30 μm FEP film (25 mm × 25 mm) was prepared and cleaned. Before etching, a layer of Cu was RF sputtered onto the surface of the FEP surface, and then O_2_, Ar, and CF_4_ gases were fed into the inductively coupled plasma (ICP) chamber to etch the surface with flow rates of 10.0, 15.0, and 30.0 standard cubic centimeter per minute. Then, a 400 W power source was used to generate the plasma, while another 100 W power source was used to accelerate the plasma ions moving to the FEP surface. After 5 min, the desired nanostructures were obtained on the Cu coated FEP surface.

### Characterization and electrical measurements of the TBJT

The current and voltage was quantitatively determined by a linear motor (Akribis-DGL-SH417), a Low Noise Current Preamplifier (SRS-SR570) and a programmable electrometer (Keithley 6514) under ambient conditions at room temperature. A DC power supply (RIGOL-DP-832) was used to supply an electrically applied voltage to the TBJT and the external circuits.

## Conclusion

In summary, we have demonstrated a tribotronic BJT (TBJT) based on a bipolar junction and a TENG in the single-electrode mode. With the sliding of the mobile triboelectric layer, a bias voltage is created, and the base current is amplified. The fabricated TBJT is used as a mechanical frequency monitoring sensor with a high sensitivity (1 μA Hz^−1^) and excellent stability. Moreover, the device can also be used as a finger-triggered active tactile switch. This work has extended emerging tribotronics to a device with simultaneously controlled voltage and current and has demonstrated that the new field may have innovative and promising potential applications in micro/nano-sensors, HMIs, wearable electronics, and other applications.

## Electronic supplementary material

Finite-element simulation and circuit

TBJT as a finger-triggered active smart tactile switch

Demonstration of the TBJT in controlling a table lamp

Demonstration of the TBJT in controlling an electric fan

Demonstration of the TBJT in controlling a household security alarm
