# Supplementary material for: Tribotronic bipolar junction transistor for mechanical frequency monitoring and use as touch switch
Source: Microsyst Nanoeng. 2018 Nov 5;4:25. doi: 10.1038/s41378-018-0026-1 (PMC6220156; doi:10.1038/s41378-018-0026-1)
Supplement: Supplementary file 1 — Finite-element simulation and circuit [file 41378_2018_26_MOESM1_ESM.doc]

**Supplementary information**


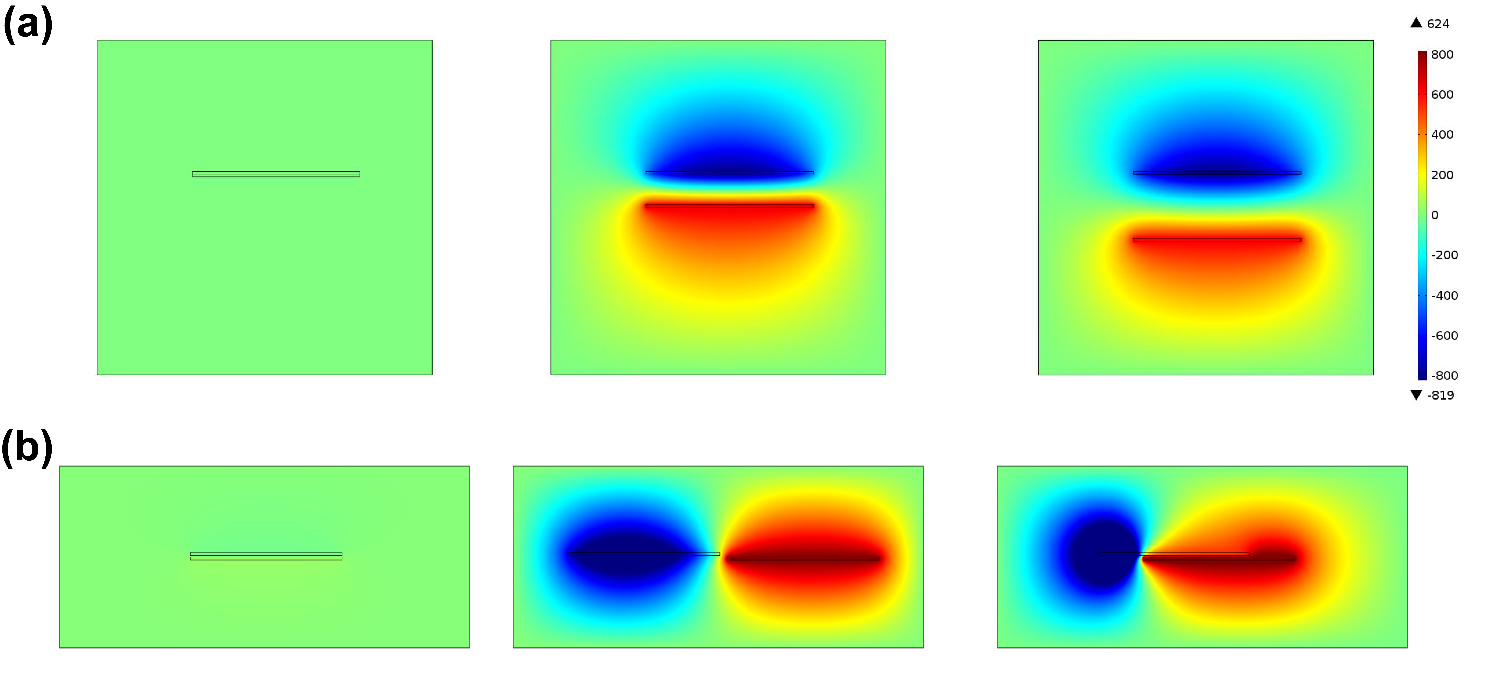


**Figure S1.** Finite-element simulation results of the potential difference between the FEP and copper films at different positions. (a, b) Electric potential distributions of the device structure with contact-separating mode and sliding mode, respectively.


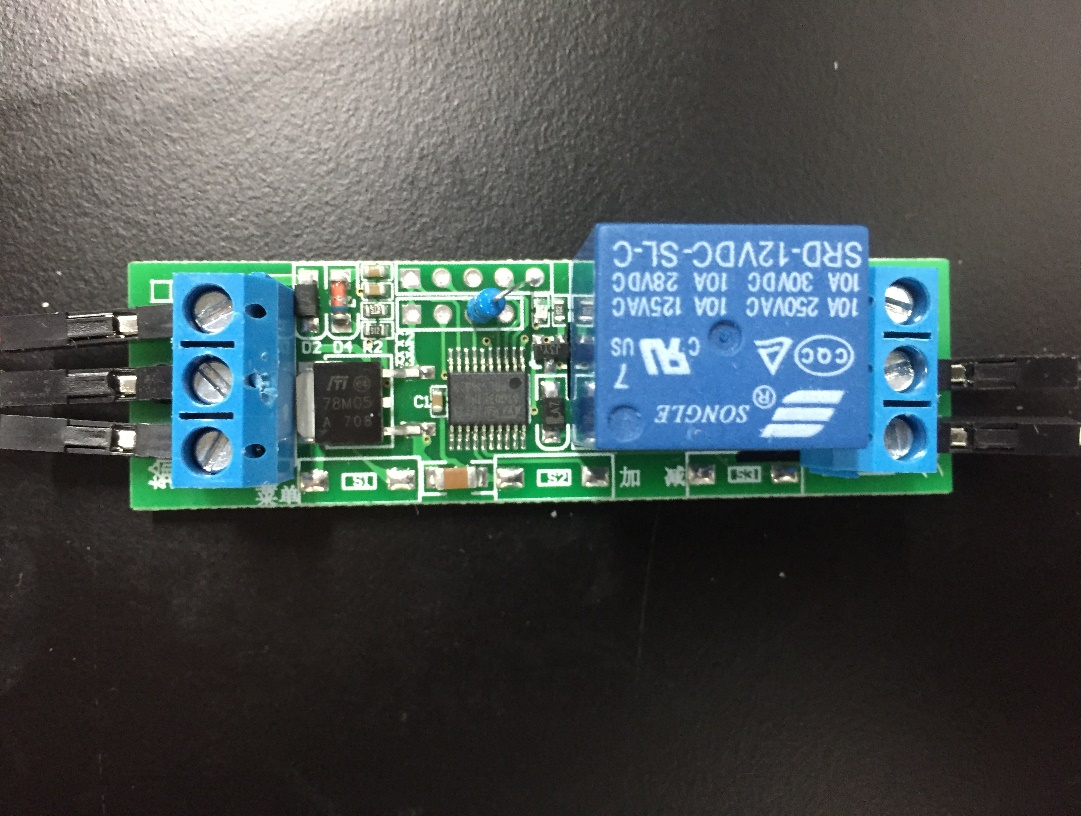


**Figure S2.** The circuit for signal conditioning.

**Video S1.** TBJT as a finger-triggered active smart tactile switch.

**Video S2.** Demonstration of the TBJT in controlling a table lamp.

**Video S3.** Demonstration of the TBJT in controlling an electric fan.

**Video S4.** Demonstration of the TBJT in controlling a household security alarm.
